# All-Inside Versus Inside-Out Meniscus Repair: Gaps in the Long-Term Current Evidence

**DOI:** 10.3390/bioengineering13010062

**Published:** 2026-01-06

**Authors:** Cariane Driad, Maika Bazebi, David Mazy, Marie-Lyne Nault

**Affiliations:** 1 CHU Sainte-Justine, 7905-3175 Côte Ste-Catherine, Montreal, QC H3T 1C5, Canada; cariane.driad@mail.mcgill.ca (C.D.); maika.bazebi@umontreal.ca (M.B.); david.mazy27@gmail.com (D.M.); 2 Department of Surgery, University of Montreal, 2900 Boul. Edouard-Montpetit, Montreal, QC H3T 1J4, Canada

**Keywords:** all-inside repair, inside-out repair, knee meniscus, meniscal repair

## Abstract

Meniscal repair has become the preferred treatment for many meniscal tears. As a result, multiple arthroscopic techniques have evolved, including the all-inside (AI) and inside-out (IO) approaches, which have been widely studied in the current literature. The present article highlights key limitations in studies reporting long-term outcomes (≥5 years), notably the heterogeneity of failure definitions and the lack of subgroup stratification by clinically relevant factors such as age, concomitant anterior cruciate ligament reconstruction (ACLR), and meniscal side (medial vs. lateral). To date, no clear superiority of the AI over the IO approach has been established. Redefining failure through multidimensional approaches that integrate structural, clinical, and patient-reported assessments will be crucial to ensure a consistent and patient-centered evaluation of repair success. Further research with robust subgroup analyses is needed to determine whether one technique confers superior long-term results in specific patient populations.

## 1. Introduction

Menisci fulfill several essential biomechanical and biological functions, including load distribution, shock absorption, reductions in joint contact stresses, passive stabilization, enhancement of joint congruity and facilitation of joint lubrication and nutrition [1,2].

When meniscal function is compromised, both biomechanical and biochemical processes are altered, leading to a loss of homeostasis within the knee joint [3,4]. Classic data following total meniscectomy demonstrate a marked reduction in tibiofemoral contact area with a corresponding increase in contact pressures, reinforcing the meniscus as a key structure for long-term joint preservation [4,5].

Meniscal tears represent one of the most common knee injuries, with reported incidences reaching up to 830 per 100,000 individuals in at-risk populations and approximately 70 per 100,000 in the general population [6,7,8]. In response to the long-term consequences of meniscal deficiency, meniscal preservation strategies have become increasingly prioritized, and meniscal repair is now widely favored over meniscectomy [9,10].

Arthroscopic repair techniques commonly used include inside-out (IO), all-inside (AI), and outside-in (OI) approaches. Traditionally, IO repair has been viewed as the gold standard due to strong fixation and versatility across tear patterns, although it requires accessory incisions and carries neurovascular risk [11,12,13]. Conversely, the AI technique was designed to minimize invasiveness by eliminating the need for additional incisions, thereby reducing surgical time and lowering the risk of neurovascular injury [11,14,15]. The OI technique is particularly advantageous for repairing the anterior horn of the meniscus and for anterior horn fixation of meniscal allografts [16,17]. Over the past two decades, advances in device technology have made the AI approach an increasingly attractive alternative to the conventional IO technique [10,13,14]. A comparative overview of AI and IO meniscal repair is presented in Table 1.

Determining which meniscal repair technique provides the most favorable outcomes remains a major focus of the contemporary literature. However, interpretation of AI versus IO comparisons is limited by two recurring methodological shortcomings: inconsistent and often narrow definitions of “failure,” and insufficient subgroup stratification in clinically meaningful populations. Accordingly, this narrative review synthesizes the current limitations in long-term outcome reporting, with particular emphasis on failure-definition heterogeneity and gaps in subgroup-based analyses.

## 2. Search Strategy and Evidence Identification

A structured literature search was performed in PubMed (MEDLINE) and Embase (Ovid) to identify comparative clinical studies evaluating AI versus IO meniscal repair. The primary objective was to capture evidence addressing mid- to long-term outcomes, with emphasis on studies reporting ≥5 years of follow-up, given that a clinically meaningful proportion of repair failures may occur beyond the early postoperative period [27].

This work was designed as a comprehensive narrative review, rather than a systematic review, with the intent to (i) map the long-term comparative evidence base and (ii) synthesize the major methodological limitations that restrict interpretation of AI versus IO outcomes. Two reviewers independently examined the literature published between 1 January 2020, and 5 September 2025, resolving any disagreements through discussion; in keeping with the narrative (non-systematic) design, this process did not follow a formal PRISMA protocol. The search terms and overarching strategy are summarized in Appendix A (Table A1 and Table A2). Study inclusion prioritized clinical comparative data and imaging-based evaluations that meaningfully inform long-term repair durability, while biomechanical studies, animal models, and purely technical reports were considered outside the scope of this synthesis. From the literature examined, a subset of well-reported studies with ≥5 years of follow-up provided especially relevant insights into long-term durability and patient outcomes. These studies are discussed in detail in this narrative synthesis and are summarized in Table 2.

Importantly, although the review emphasizes recent publications, relevant post-2020 systematic reviews and meta-analyses were also considered when they contributed long-term comparative estimates, even when their included primary studies pre-dated 2020. This approach was used to avoid an overly narrow evidence base for ≥5-year outcomes and to allow contextual interpretation of how outcome definitions and device generations may have evolved over time. Throughout the synthesis, particular attention was given to (i) the definition of failure applied in each study, (ii) the generation and type of AI device used (e.g., early resorbable rigid implants vs. modern non-resorbable suture-based systems), and (iii) whether key clinical subgroups were analyzed separately.

## 3. Definition of Failure

Heterogeneity in failure definitions largely reflects the tendency for studies to operationalize “success” using a single outcome domain, such as revision surgery, clinical symptoms, patient-reported outcome measures (PROMs), radiologic findings, or second-look arthroscopy. These endpoints capture different dimensions of postoperative recovery and carry distinct strengths and limitations; the following sections summarize the principal failure criteria used in the literature and discuss their implications for interpreting repair durability.

### 3.1. Revision Surgery

The objectivity of reoperation criteria makes it an appealing and easily quantifiable measure [10,20,28]. However, some patients may undergo subsequent surgery for reasons unrelated to the integrity of the meniscal repair, while others with persistent symptoms and a potential surgical indication may avoid reoperation altogether, potentially leading to an underestimation of the true failure rate. Furthermore, whether the need for revision surgery genuinely reflects clinical failure remains debated, as the indication for reoperation is often subjective and varies between surgeons. Persistent symptoms may occur despite the absence of structural failure on imaging; however, in the absence of a surgical indication, these cases are often excluded from failure definitions based exclusively on revision surgery [29].

### 3.2. Clinical Symptoms

Several studies evaluate surgical success based on clinical symptoms such as persistent knee pain, swelling, locking or mechanical dysfunction [29]. Some authors rely on the Barrett criteria, which assess joint line tenderness, pain during hyperextension or flexion, swelling and locking. Compared with revision surgery, this approach offers a more comprehensive and standardized assessment of knee function [12,15]. Its advantage lies in encompassing patients who continue to experience symptoms despite not undergoing reoperation. While some authors have reported a direct correlation between clinical symptoms and anatomical failure, others have shown that symptoms may persist even in the absence of abnormalities on MRI or arthroscopy [30]. In fact, synovial and meniscal inflammation has been shown to negatively influence pain and knee symptoms in the setting of meniscal injury, both preoperatively and during the early postoperative period, even when synovitis is only microscopic [31]. It may also contribute to mechanical and structural alterations within the meniscal tissue and increase the risk of developing or accelerating OA [32]. This discrepancy highlights the potential mismatch between clinical presentation and structural findings, emphasizing the need for a multidimensional approach to evaluating meniscal repair success [29].

### 3.3. PROMs

Moreover, PROMs are commonly used to evaluate the success of meniscal repair, as they capture the patient’s perspective on symptoms and functional recovery. Common knee-specific PROMs include the International Knee Documentation Committee (IKDC) score, the Knee Injury and Osteoarthritis Outcome Score (KOOS), the Tegner Activity Scale and the Lysholm scale. The Tegner–Lysholm system, which combines both the activity and symptom-based components, provides a complementary assessment of function and activity level [29,33,34,35,36]. The validity of PROMs in defining failure after meniscal repair remains debated. Some studies have reported a positive association, where higher PROMs scores correlate with favorable anatomical findings and vice versa [37,38]. Others, however, highlight inconsistencies: patients may report satisfactory function despite an anatomical retear on imaging or arthroscopy, while others with low scores show no detectable abnormality [29,33,39]. Several factors may account for this discrepancy. PROMs are inherently subjective and can be influenced by pain tolerance, patient expectations, mental health or changes in activity level. In addition, heterogeneity across scoring systems complicates interpretation: for example, the Tegner-Lysholm scale assesses different domains than the KOOS or IKDC, and each employs distinct scoring thresholds for clinically meaningful change. To address these limitations, some authors have proposed using the WOMET (Western Ontario Meniscal Evaluation Tool), specifically designed to assess meniscus-related symptoms [29,40]. Despite their limitations, PROMs remain valuable tools for assessing functional recovery. Most authors recommend using them in conjunction with objective measures such as imaging or arthroscopy to provide a more comprehensive evaluation of meniscal repair outcomes [29,33].

### 3.4. Radiologic Outcomes

Magnetic resonance imaging (MRI) is widely recognized as the preferred non-invasive modality for assessing acute meniscal injuries, demonstrating high sensitivity, specificity and diagnostic accuracy, typically ranging from 85% to 90% [41]. However, interpretation in the postoperative setting can be challenging, as signal alterations, granulation tissue and fibrosis may mimic recurrent tears, particularly within the first three postoperative months. When used to evaluate repaired menisci, conventional MRI has shown an accuracy ranging from 57% to 80% in detecting retears, reflecting the difficulty of differentiating between true failure and normal healing changes [41,42]. Recent advances, including higher magnetic field strengths and three-dimensional isotropic proton density-weighted sequences, have improved diagnostic confidence in the postoperative evaluation of meniscal repairs [41]. While postoperative MRI remains the preferred non-invasive modality to assess meniscal repair integrity and to evaluate suspected retears, most long-term studies use MRI primarily for repair status rather than for standardized assessment of joint preservation. Long-term radiological endpoints that are central to the rationale for meniscal preservation, such as cartilage status, meniscal extrusion, subchondral changes, and radiographic OA progression (e.g., joint-space narrowing and Kellgren–Lawrence grading), are inconsistently captured and rarely incorporated into outcome frameworks [14,21,41,43]. Accordingly, imaging-based definitions of “failure” after meniscal repair should not be limited to confirming or excluding a retear; when feasible, they should integrate standardized measures of structural joint degeneration alongside clinical and PROMs to better reflect the long-term goals of meniscal preservation.

### 3.5. Second-Look Arthroscopy

Second-look arthroscopy remains the most direct and reliable method for assessing meniscal healing and is widely regarded as the gold standard for diagnosing recurrent meniscus tears [14,41,42]. It allows for visual confirmation of tissue continuity, suture integrity, and surface morphology [14]. During second-look evaluation, healing is typically categorized as complete, partial, or absent, based on the continuity of the meniscal substance and its integration with surrounding tissue [14,19]. Although it provides valuable anatomical information, second-look arthroscopy is invasive and is therefore reserved for specific situations, such as symptomatic patients, planned hardware removal, or concomitant surgical procedures. Moreover, using this method solely to assess meniscal repair integrity is ethically debatable, as it carries all the inherent risks of surgery without offering a clear clinical benefit [14,21]. In most studies, MRI and second-look arthroscopy are used as complementary tools. MRI provides a noninvasive overview of the meniscal repair status, while arthroscopy offers definitive confirmation when imaging results or clinical symptoms are inconclusive [14,19,41]. Defining failure based on these objective assessments contributes to a more standardized and reproducible evaluation of meniscal repair outcomes [14,19,41].

### 3.6. Multidimensional Approaches

Taken together, these findings underscore the lack of a universally accepted definition of meniscal repair failure. Each assessment method, whether based on revision surgery, clinical symptoms, imaging or arthroscopic evaluation, offers distinct advantages and inherent limitations. While revision surgery provides objective data, it may underestimate the true failure rates; clinical and PROM-based evaluations better capture functional outcomes but remain subjective; and imaging and arthroscopy yield valuable structural information, although their correlation with symptoms is often inconsistent. Consequently, a multidimensional approach that integrates structural, clinical and patient-reported assessments is essential for accurately characterizing meniscal repair outcomes. In line with this, Kamaci et al. [29] recently proposed a combined model that offers a more comprehensive and patient-centered framework for future research and clinical evaluation. Their approach defines “combined failure” across three dimensions: an anatomical component, referring to imaging or second-look arthroscopic evidence of retear; a clinical component, characterized by persistent symptoms or abnormal physical examination findings; and a functional component, indicated by poor patient-reported outcomes or dissatisfaction despite evidence of healing [29].

## 4. All-Inside vs. Inside-Out

### 4.1. Comparable Outcomes & Failure Heterogeneity

Across the long-term literature published since 2020, including original comparative studies and recent meta-analyses that draw on earlier work, definitions of meniscal repair failure vary substantially, ranging from revision surgery endpoints to imaging-confirmed retears [10,18,21]. A study-by-study mapping of the failure definitions used in the studies that form the basis of our analysis is provided in Table 3.

Only a portion of studies incorporated anatomical criteria such as second-look arthroscopy or MRI to define failure [14,21]. When failure was assessed using these direct visualization or imaging criteria, the available evidence indicated no significant difference in mid-term to long-term repair survival between the conventional IO technique and the AI technique [14,21]. Beyond the evaluation of retears on postoperative MRI, radiological outcomes related to long-term joint preservation were inconsistently reported across studies comparing AI and IO meniscal repair. Only Petersen et al. [14] provided radiographic assessment of osteoarthritic changes, reporting generally mild degenerative progression after repair but without a control group and without differentiating between surgical techniques. Consequently, current evidence does not permit any meaningful radiological comparison of long-term joint preservation between AI and IO repairs. Standardized long-term imaging protocols incorporating radiographic OA grading and MRI-based assessment of cartilage are needed to determine the true protective effect of each technique.

The systematic review by Petersen et al. [14] analyzed twelve studies, nine of which defined failure as an arthroscopically confirmed retear and one of which combined arthroscopy with MRI confirmation. Altogether, the review included 245 IO repairs, 176 AI repairs using both resorbable and non-resorbable devices, and a subset of 94 performed with modern non-resorbable AI implants. Reported failure rates ranged between roughly 22% and 30%, and no statistically significant difference was found between the two techniques. The authors emphasized that the long-term durability of flexible AI suture devices appears comparable to that of traditional IO sutures when outcomes are evaluated by second-look arthroscopy or MRI [14]. Despite these findings, the criteria used to define failure across the included studies remained inconsistent. Although most studies reported functional outcomes using PROMs such as the KOOS, Lysholm, or subjective IKDC scores, these instruments were neither used to compare functional performance between repair techniques nor included as part of the failure definition but were instead analyzed as secondary outcomes. This approach reflects a broader pattern in the meniscal repair literature, where anatomical healing is often prioritized over patient-relevant functional recovery.

Moreover, a systematic review and meta-analysis by Nepple et al. [18] defined failure more broadly, as the recurrence of clinical symptoms or the need for any subsequent meniscal reintervention. Based on this definition, the authors analyzed 27 studies (1612 patients; 1630 repairs; mean follow-up of 10.8 years) and found comparable long-term failure rates between IO repairs (14.2%) and modern AI techniques (15.8%) [18]. In this study, the definition of failure is problematic because it is broadly framed. The review does not clarify how these criteria were applied or whether clinical symptoms and reintervention were considered equivalent indicators of failure. This is particularly concerning given that clinical presentation and the need for reintervention do not necessarily align. As a result, comparing failure rates across studies that rely on non-equivalent definitions reduces interpretability and weakens the overall strength of the conclusions; moreover, the conclusions themselves may be inherently limited when “success” is inferred from a single outcome domain (e.g., anatomical healing or functional recovery) rather than an integrated assessment that captures the full clinical relevance of meniscal repair.

The studies included in the reviews by Petersen et al. [14] and Nepple et al. [18] underscore a key limitation in the current literature: failure is often defined using narrow or non-equivalent criteria that do not fully capture clinically meaningful recovery. These inconsistencies raise methodological concerns. Anatomical healing, symptomatic improvement, and the decision to reintervene reflect different dimensions of postoperative recovery. Treating these outcomes as interchangeable, or privileging structural findings over functional recovery, can obscure clinically meaningful differences between repair techniques. A more comprehensive definition integrating anatomical, clinical, and functional dimensions, as suggested by Kamaci et al. [29] would provide a more meaningful assessment of true repair success.

In contrast, Lamba et al. [21] applied a long-term and relatively stringent failure framework and reported no meaningful difference between techniques. In their comparative cohort study of 63 bucket-handle meniscal tears (BHMTs), 37 patients underwent IO repair and 26 underwent AI repair [21]. Failure was assessed using a robust composite definition integrating objective structural endpoints (retear confirmed on second-look arthroscopy or symptomatic retear identified on imaging) with PROMs at final follow-up, namely the Tegner Activity Scale and the IKDC. This multidimensional approach was designed to reflect not only anatomical integrity but also long-term functional and clinical outcomes. At a mean follow-up of approximately 11 years, overall survivorship was 63%, with no meaningful differences between repair techniques. Ten-year survival was 70% in the AI group and 60% in the IO group, with no statistically significant difference. Time to failure and PROMs scores were comparable between groups, and no patient required subsequent meniscectomy or conversion to total knee arthroplasty [21].

Conversely, Schweizer et al. [10], reported significantly lower failure rates with the IO technique compared with the AI approach. In their systematic review and meta-analysis of 12 studies (864 patients) with a minimum follow-up of five years, IO repairs demonstrated a lower “failure” rate than AI repairs at five years (5.6% vs. 22.3%; *p* = 0.009). However, failure was defined solely as revision surgery, an endpoint that may underestimate true failure by excluding symptomatic retears managed nonoperatively and structural failures detected on imaging or second-look arthroscopy without reoperation, while also potentially overestimating failure if revisions were undertaken primarily for persistent symptoms in the absence of clearly documented structural retear, since revision indications were not consistently defined or transparently reported across included studies. Interpretation is further limited by device heterogeneity, with most studies relying on early-generation AI implants [10].

Several studies have suggested that the IO technique may yield superior outcomes compared with the AI approach when early-generation AI devices were used. Nepple et al. [18] reported that these early devices were associated with markedly higher failure rates (30.2%), while the pooled failure rate across modern repair techniques was substantially lower (19.5%). Likewise, Petersen et al. [14] observed device-specific variations in re-rupture rates, with 22.3% for the FAST-FIX™ system, representative of contemporary suture-based AI devices, and 48% for RapidLoc, an early-generation bioabsorbable device [11,13,14].

Even though this review primarily targeted literature published after 2020, meta-analyses that incorporated pre-2020 data were included, as excluding them would have left too few studies for a meaningful evaluation of long-term outcomes. Ultimately, the goal was to provide a clear overview of what has been published in the past five years, distinguishing truly novel findings from established knowledge. In doing so, the evolution of meniscal repair devices itself emerged as a key observation of this review. Therefore, identifying which device and generation were used in each study is essential for accurately interpreting which repair technique yields superior outcomes. Future research should consistently report the specific devices and their corresponding generations to allow for more precise comparison and interpretation of results.

### 4.2. Subgroups Differences

Beyond the type of device used, potential effect modifiers, including tear laterality, patient age, and concomitant anterior cruciate ligament reconstruction (ACLR), should also be considered. Although most studies report broadly comparable outcomes between AI and IO repairs, several authors have suggested subgroup-specific differences; however, these signals remain difficult to interpret because subgroup analyses are often underpowered, inconsistently reported, and based on heterogeneous outcome definitions and follow-up protocols. Potential effect modifiers reported in the long-term AI versus IO meniscal repair literature, including meniscal side (medial vs. lateral), patient age and concomitant ACLR are summarized in Table 4.

### 4.3. Laterality of Meniscus Repair

Regarding the lateralization of the tear, three out of the four studies examined the influence of tear location on outcomes, with findings that were not entirely consistent. Schweizer et al. [10], in their review, included a total of 310 medial and 161 lateral menisci across five of the twelve studies that addressed tear lateralization. The pooled failure rates were 24.4% for medial repairs and 19.5% for lateral repairs. Subgroup analysis showed no statistically significant difference between medial and lateral meniscus repair failure rates. This finding contrasts with the results of Nepple et al. [18], who, in their meta-analysis, reported that medial repairs demonstrated higher failure rates than lateral repairs. In total, 678 medial and 328 lateral meniscal repairs were analyzed. The pooled failure rate for medial repairs was 23.9%, which was significantly higher than the 12.6% observed for lateral repairs. Similarly, Lamba et al. [21] found that medial meniscal repairs were associated with an increased risk of surgical failure. Both studies attributed this difference to the biomechanical and anatomical characteristics distinguishing the medial from the lateral meniscus, the medial meniscus being less vascularized and more rigidly anchored to the tibial plateau [44].

Nonetheless, neither study specified whether the surgical technique influenced repair outcomes. It therefore remains unclear whether the AI technique for medial meniscus repairs results in significantly poorer outcomes compared to the IO technique and vice versa, as no subgroup analysis was conducted. This concern aligns with the findings of Borque et al. [20], who reported a failure rate in patients with a medial meniscal tear repaired with the AI technique that was almost 8 times higher at any point in time compared with a lateral meniscal tear repaired with the IO technique in an elite athlete population. These findings underscore the importance of future research distinguishing between medial and lateral meniscus tears when assessing repair outcomes in the general population, with particular attention to whether the relative performance of the AI and IO techniques varies according to tear location.

### 4.4. Influence of Age

Moreover, regarding the influence of patient age, earlier studies suggested that age had little influence on meniscal repair outcomes [45,46,47,48,49]. However, while some reports indicated that pediatric patients tend to exhibit lower failure rates due to their greater intrinsic healing potential [18], others proposed that older patients may experience fewer failures because of reduced physical activity levels [14,20]. Petersen and colleagues observed that adolescents and young adults were more susceptible to re-rupture, likely owing to higher post-operative activity [14]. Similarly, Lamba et al. [21] identified increasing age as a significant correlate of decreased failure rates following meniscal repair. In contrast, Nepple et al. [18] found no significant difference in failure rates between cohorts in which most patients were younger than 18 years and adult populations. They suggested that although the superior healing potential of pediatric patients could confer an advantage, this benefit may be offset by their higher activity levels, which increase the risk of reinjury. Consistent with this complexity, Fauré et al. [28] reported relatively high mid- to long-term survival in a purely pediatric and hypothesized that these outcomes were related to their use of standardized IO vertical non-absorbable sutures. This observation supports the notion that optimized repair technique may partially counterbalance the elevated failure risk traditionally ascribed to younger, more active patients. Collectively, these findings underscore the complex interplay between age, biological healing potential, postoperative activity levels, and meniscal repair technique in determining meniscal repair success. None of the available studies have stratified outcomes simultaneously by repair technique, patient age and activity level. Although short-term evidence indicates that postoperative activity levels are comparable between the IO and AI repair techniques [23], some studies have suggested a potential advantage of the AI approach in facilitating an earlier return to sport [24,25]. Robust long-term data are needed to better define the relationship between repair technique, patient age, and sustained activity levels, ultimately helping to tailor surgical strategies to individual patient profiles and activity demands.

### 4.5. Status of the Anterior Cruciate Ligament (ACL)

Although early studies suggested that the biological environment created during ACLR might enhance meniscal healing, the present review did not identify concomitant ACLR as a protective factor against long-term meniscal repair failure. This finding contrasts with the long-standing concept in orthopedic surgery that concomitant ACLR promotes superior meniscal healing [50,51,52,53]. Across the included systematic reviews and meta-analyses, long-term repair survivorship was comparable between isolated meniscal repairs and those performed with concurrent ACLR. Interpretation of these findings is limited by substantial heterogeneity in repair devices, surgical techniques, and failure definitions, as well as by limited reporting on biologic augmentation strategies and tear characteristics [10,14,18,51]. Despite these methodological constraints, the results are in line with some large-scale, long-term studies that have also failed to demonstrate a statistically significant protective effect of concomitant ACLR on meniscal repair outcomes [24,51]. The lack of a detectable protective effect of ACLR may reflect the evolution of meniscal repair techniques. With the widespread adoption of flexible, suture-based all-inside devices and the increasing use of biologic enhancement strategies such as marrow venting, the biological advantage historically attributed to ACLR may have become less pronounced [24,48,50,51,54]. However, none of the included studies evaluated whether the outcomes of meniscal repair techniques differed according to ACL status.

## 5. Conclusions

Current evidence suggests that both the AI and IO meniscal repair techniques provide comparable mid-to-long-term survival and functional outcomes when modern suture-based devices are used. Divergences observed across studies appear largely attributable to differences in device generation and surgical indication rather than to the repair technique itself. While the IO approach may still offer technical advantages in select cases, the evolution of flexible, low-profile AI systems has substantially narrowed this gap. Redefining failure through more multidimensional approaches that integrate structural, clinical, and patient-reported assessments will be crucial to ensure consistent and patient-centered evaluation of repair success. Contemporary data support the notion that with appropriate patient selection and modern instrumentation, both techniques can achieve durable and clinically meaningful repair success. Future studies stratifying patients by tear location, patient age and concomitant ACLR would be valuable to delineate which repair technique provides superior long-term outcomes.

## Figures and Tables

**Table 1:** Comparative Overview of Repair Techniques: AI vs. IO.

Parameter	AI Repair Early-Generation Devices	AI Repair Modern Devices	IO Repair	Key References
Repair type	Rigid/bioabsorbable implants (e.g., RapidLoc)	Flexible non-resorbable suture-based systems (e.g., FasT-Fix)	Non-resorbable sutures tied over capsule	Nepple et al. (2022) [18] Petersen et al. (2022) [14]
Biomechanical stability	Lower fixation strength (rigid implants)	Comparable fixation to sutures/IO	High stability due to suture strength	Zantop et al. (2005) [13] Fillingham et al. (2017) [19]
Patient/tear type specificity	Higher failure risk in medial tears/high-demand cohorts	Advantage in BHMT/posterior horn	Advantage in complex/medial tears (technique versatility)	Borque et al. (2023) [20] Lamba et al. (2024) [21] Nepple et al. (2022) [18] Marigi et al. (2022) [22]
Clinical outcomes	Higher failure rates	Comparable to IO	Comparable to modern AI	Nepple et al. (2022) [18] Schweizer et al. (2022) [10] Petersen et al. (2022) [14] Lamba et al. (2024) [21]
Risk profile	Implant-related complications	Reduced implant-related complications	Nerve injury risk from dissection (saphenous/peroneal nerves)	Grant et al. (2012) [23] Fillingham et al. (2017) [19]
Rehabilitation considerations	Similar protocols	Similar, some reports suggest earlier RTS	Similar protocols	Totlis et al. (2021) [24] Migliorini et al. (2023) [25]
Technical demand	Mild (no accessory incision)	Very mild (simplified instrumentation)	More technically challenging due to accessory incisions	Marigi et al. (2022) [22] Grant et al. (2012) [23]
Operative time	Shorter	Shorter	Longer due to posteromedial/posterolateral dissection	Vint et al. (2021) [26] Grant et al. (2012) [23]
Cost	Higher due to implants	Higher due to implants	Lower (suture-based)	Marigi et al. (2022) [22] Yılmaz et al. (2016) [12]

AI All-inside, IO Inside-out, BHMT Bucket-handle meniscal tear, RTS Return to sport.

**Table 2:** Characteristics of Included Studies Comparing AI vs. IO Meniscal Repair.

Author (Year)	Study Design	Follow Up (Years)	No. of Repairs & Surgical Technique (AI/IO)	Repair Device	Main Result (AI vs. IO)
Petersen et al. (2022) [14]	Systematic review (12 studies included)	≥7	270 All-inside	176 EGD 94 MD	No significant difference in failure with modern AI device
			245 Inside-out	N/A	
Schweizer et al. (2021) [10]	Systematic review & meta-analysis (12 studies included)	≥5	464 All-inside	Not specified	Lower failure in IO than AI at 5 years
			229 Inside-out	N/A	
Nepple et al. (2022) [18]	Systematic review & meta-analysis (27 studies included)	≥5	664 All-inside	402 EGD 262 MD	No significant difference in failure with modern AI device
			387 Inside-out	N/A	
Lamba et al. (2024) [21]	Comparative cohort (bucket-handle tears)	≥9	26 All-inside	0 EGD 26 MD	No significant difference in failure with modern AI device
			63 Inside-out	N/A	

Repair devices are specified for all-inside technique. AI All-inside, IO Inside-out, N/A Not applicable, EGD Early generation device, MD Modern device.

**Table 3:** Failure Definitions in Long-Term Comparative Studies of AI Versus IO Meniscal Repair.

Author (Year)	Definition of Failure Used	PROMs Included	Radiologic OA
Petersen et al. (2022) [14]	Second look arthroscopy +/− MRI	Not included in failure definition *	Mild OA changes in some cohorts; No AI vs. IO comparison
Schweizer et al. (2021) [10]	Revision surgery	Not included in failure definition	Not assessed
Nepple et al. (2022) [18]	Recurrent symptoms OR any reintervention	Not included in failure definition	Not assessed
Lamba et al. (2024) [21]	Symptomatic retear + imaging/second look arthroscopy + PROMs at final follow-up	Tegner activity scale Subjective IKDC	Not assessed

* PROMs were reported but were not included in the failure definition, as they were used solely as secondary outcomes. PROMs Patient-Reported Outcome Measures, OA Osteoarthritis, AI All-inside, IO Inside-out, MRI Magnetic resonance imaging, IKDC International Knee Documentation Committee.

**Table 4:** Summary of subgroup-level evidence and technique-by-subgroup gaps in long-term AI versus IO meniscal repair outcomes.

Potential Modifier	Included Studies (Year)	Key Findings	AI vs. IO Technique-by-Subgroup Evidence
Meniscal side (medial vs. lateral)	Petersen et al. (2022) [14]	NA	Unclear if AI vs. IO significantly differ according to tear laterality (medial vs. lateral).
	Schweizer et al. (2021) [10]	No significant difference in failure or reoperation rates between medial and lateral tears.	
	Nepple et al. (2022) [18]	Medial tears associated with higher surgical failure risk than lateral tears.	
	Lamba et al. (2024) [21]	Medial tears associated with higher surgical failure risk than lateral tears.	
Age	Petersen et al. (2022) [14]	Increasing age correlated with lower failure risk.	No study designed to test a formal age and technique interaction.
	Schweizer et al. (2021) [10]	Age effect not specifically analyzed.	
	Nepple et al. (2022) [18]	No clear difference in failure	
	Lamba et al. (2024) [21]	Increasing age correlated with lower failure.	
Concomitant ACLR	Petersen et al. (2022) [14]	No clear difference in failure	No study assessed the potential interaction between ACL status and repair technique.
	Schweizer et al. (2021) [10]	No clear difference in failure	
	Nepple et al. (2022) [18]	No significant difference	
	Lamba et al. (2024) [21]	No significant difference	

AI All-inside, IO Inside-out, NA Not Assessed, ACL Anterior Cruciate Ligament, ACLR Anterior Cruciate Ligament Reconstruction.

## Data Availability

The original contributions presented in this study are included in the article. Further inquiries can be directed to the corresponding author.

## Abbreviations

The following abbreviations are used in this manuscript:

IO	inside-out
AI	all-inside
OI	outside-in
ACLR	anterior cruciate ligament reconstruction
PROM	Patient-Reported Outcome Measures
BHMT	bucket-handle meniscal tears
IKDC	International Knee Documentation Committee
KOOS	Knee Injury and Osteoarthritis Outcome Score
WOMET	Western Ontario Meniscal Evaluation Tool

## Appendix A

### Search Strategy

A literature search was conducted on 5 September 2025, in the databases PubMed (MEDLINE) and Embase (Ovid) with the assistance of an academic librarian (Philippe Dodin, Bibliothèques du CHUSJ et du CRME). The search strategy aimed to identify studies focusing on meniscal surgery, including repair and meniscectomy techniques, with an emphasis on “all-inside,” “inside-out,” and “outside-in” approaches. The search was limited to human studies published between 1 January 2020, and 5 September 2025.

**Table A1:** PubMed Search Strategy.

Step	Query	Results
#1	“Meniscus/surgery”[Majr] OR “Tibial Meniscus Injuries/surgery”[Majr]	3751
#2	((meniscus*[TI] OR meniscal[TI] OR menisci*[TI]) NOT (Tear meniscus height*[TI] OR meniscometry[TI] OR dry eye[TI])) OR ((meniscus*[OT] OR meniscal[OT] OR menisci*[OT]) NOT (Tear meniscus height*[OT] OR meniscometry[OT] OR dry eye[OT]))	11,558
#3	Surgical Procedures, Operative[Majr] OR surg*[TI] OR sutur*[TI] OR operat*[TI] OR repair*[TI] OR “inside out”[TI] OR “all inside”[TI] OR “outside-in”[TI] OR meniscectom*[TI] OR resect*[TI] OR arthroscop*[TI] OR transplant*[TI] OR allograft*[TI] OR replacement*[TI]	3,479,398
#4	Exclusion of animal and cadaveric studies using the NOT operator	5,926,440
Final combination	(((#1 OR (#2 AND #3)) NOT #4) AND 2020:2025[pdat])	1974

**Table A2:** Ovid Embase Search Strategy.

Step	Query	Results
1	Exp *knee meniscus/su OR *knee meniscus rupture/su OR exp *meniscal surgery/	5520
2	(meniscus* OR meniscal OR menisci*) NOT (Tear meniscus height* OR meniscometry OR dry eye).ti,kw	12,823
3	Exp *surgery/OR (surg* OR sutur* OR operat* OR repair* OR “inside out” OR “all inside” OR “outside-in” OR meniscectom* OR resect* OR arthroscop* OR transplant* OR allograft* OR replacement*).ti,kw	4,046,180
4	Exclusion of animal and cadaveric studies	6,272,414
5	(1 OR (2 AND 3)) NOT 4	7460
6	Excluded “conference abstracts” and “clinical trial (clinicaltrials.gov)”	793
Final combination	5 NOT 6, limited to 2020–Current	1986
